# Biocompatibility and *in vivo* osteogenic capability of novel bone tissue engineering scaffold A-W-MGC/CS

**DOI:** 10.1186/s13018-014-0100-9

**Published:** 2014-12-12

**Authors:** Chen Li, Guo-Xian Wang, Zheng Zhang, Dan-Ping Liu

**Affiliations:** Biobank, the First Affiliated Hospital of Liaoning Medical University, Jinzhou, 121001 China; Department of Pharmacology, Liaoning Medical University, Jinzhou, 121000 China; Department of Orthopaedic Surgery, First Affiliated Hospital of Liaoning Medical University, No. 2 Wuduan Renmin Street Guta District, Jinzhou, 121001 China

**Keywords:** Bone tissue engineering, A-W-MGC/CS, Bone defect, Repair

## Abstract

**Background:**

This study aims to investigate the biocompatibility and *in vivo* osteogenic capability of the novel bone tissue engineering scaffold apatite-wollastonite-magnetic glass ceramic/chitosan (A-W-MGC/CS).

**Methods:**

Rabbit bone marrow stromal cells (BMSCs) were transfected with adenovirus-human bone morphogenetic protein-2-green fluorescent protein (Ad-hBMP2-GFP). The transfected BMSCs were then inoculated onto the scaffold material A-W-MGC/CS to construct tissue-engineered bone. The attachment and proliferation of BMSCs were observed by scanning electron microscopy (SEM) and 3-(4,5-dimethylthiazol-2-yl)-2,5-diphenyltetrazolium bromide (MTT) detection, respectively. Rabbit models of bone defects were established and divided into three groups. Experimental group 1 was implanted with prepared tissue-engineered bone. Experimental group 2 was implanted with A-W-MGC/CS without transfected BMSCs. The blank group was injected with transfected BMSCs, without implantation of any scaffold. In the 12th week after surgery, the repair of bone defect was observed by X-ray examination, and histological observations of the area of bone defect were performed.

**Results:**

A-W-MGC/CS resulted in good BMSC attachment and had no obvious effects on cell proliferation. In experimental group 1, good repair of bone defect was observed, and the scaffold material degraded completely. In experimental group 2, new bone was formed, but its quality was poor. In the blank group, there was mainly filling of fibrous connective tissues with no observable bone defect repair.

**Conclusion:**

A-W-MGC/CS possesses good biocompatibility and *in vivo* osteogenic capability for bone defect repair.

## Background

The repair of bone defects is a challenging problem in the medical field. Autogenous bone grafting is the most commonly used method, but the bone supply is limited and some complications may occur in the donor area [1,2]. As allogeneic bone creates antigenicity, allogeneic bone grafting often fails because of intense immune rejection, especially for the grafting of large bone fragments [3,4]. In recent years, the development of tissue engineering technology has allowed the induction of cell differentiation, proliferation, and implantation on biological materials, in turn promoting the repair of a wide range of bone defects [5-7]. The three-dimensional complex composed of cells and biological materials lies at the core of bone tissue engineering, and the scaffolding material is one of the basic elements [8]. Composite materials can comprehensively embody the advantages of various materials and have achieved good effects in practical applications. Apatite-wollastonite-magnetic glass ceramic (A-W-MGC) [9] is a new composite scaffold in bone tissue engineering with appropriate porosity, good biocompatibility, and superior mechanical strength. Apatite-wollastonite-magnetic glass ceramic/chitosan (A-W-MGC/CS) is compositely prepared from A-W-MGC and chitosan, which can further enhance the biocompatibility and improve the pore structure and degradation characteristics of the scaffold. In addition, it can provide suitable pores for cell growth and adhesion and is suitable for the construction of tissue-engineered bone. In this study, the biocompatibility of A-W-MGC/CS and the *in vivo* osteogenic capability of tissue-engineered bone constructed from A-W-MGC/CS were investigated.

## Material and methods

### Isolation and culture of bone marrow stromal cells

This study was conducted with approval from the Animal Ethics Committee of Liaoning Medical University. Bone marrow (4 mL) was drawn from the proximal tibia of a Japanese white rabbit (clean grade, 1.5–2 months old, male or female, provided by the Experimental Animal Center of Liaoning Medical University) and was added to a sterile flask. Then 4 mL of DMEM containing 10% fetal bovine serum (FBS) was added to the flask (DMEM and FBS were provided by Gibco Inc., Montana, USA). After repetitive beating, 2 mL of the mixture was transferred to another flask, followed by the addition of 2 mL of culture medium. After 5 days, the culture medium was replaced with fresh medium. The growth of bone marrow stromal cells (BMSCs) was observed. When 80% of adherent cells were fused, the primary culture was closed and subculture was performed. The third generation of cells was collected for use.

### Transfection of BMSCs with adenovirus

The third generation of BMSCs (5 × 10^5^ cells/bottle) was transfected by adenovirus-human bone morphogenetic protein-2-green fluorescent protein (Ad-hBMP2-GFP) (previously prepared in our laboratory) with a multiplicity of infection (MOI) of 100. The transfection results were observed at 24 h and 48 h under a fluorescence microscope. Flow cytometry (exciting light, 488 nm; receiving light, 530 nm) was performed for cell counting. The results were expressed as transfection rate and mean fluorescence intensity of GFP-positive cells.

### Construction of tissue-engineered bone

A-W-MGC/CS (15 mm × 5 mm × 5 mm, provided by Professor Zhou Dali from the Department of Inorganic Non-metallic Materials of Sichuan University, Chengdu, China) was disinfected using ethylene oxide. Before use, A-W-MGC/CS was washed three times with PBS and then immersed in DMEM without FBS for 7 days. Finally, the culture medium was removed and A-W-MGC/CS was naturally dried in an incubator. A suspension of transfected BMSCs (5 × 10^6^ cells/mL) was inoculated onto A-W-MGC/CS to prepare the tissue-engineered bone, followed by transfer into a 24-well plate for incubation. After 2 h, the tissue-engineered bone was turned over. After 4 h, 1.5 mL of DMEM containing 20% FBS was added to each well for 3 days of culture. The culture medium was replaced with fresh medium once a day.

### Establishment of rabbit models of bone defect

Eighteen Japanese white rabbits (clean grade, 2–3 months old, 1.5–2 kg, provided by the Experimental Animal Center of Liaoning Medical University) were enrolled in the establishment of bone defect models. A longitudinal incision (length of 3 cm) was made in the anteriomedialis of the left and right forearms. The skin and fascia were incised, followed by layer-by-layer separation of muscle, vessel, and nerve. The middle radius was fully exposed. A 1.5-cm bone defect was made, and the periosteum in the defect area was carefully removed. The rabbit models of bone defect were established and divided into three groups. After washing the incision with normal saline, experimental group 1 was implanted with A-W-MGC/CS with transfected BMSCs. Experimental group 2 was implanted with A-W-MGC/CS without transfected BMSCs. The blank group was injected with transfected BMSCs without implantation of any scaffold. Finally, the incision was closed layer-by-layer. Each rabbit was reared in a separate cage without intraoperative and postoperative fixation.

### MTT detection

The third generation of BMSCs was inoculated in a 96-well plate at a density of 2,000 cells/well. Culture medium (200 μL) immersing A-W-MGC/CS was added to 21 wells (experimental group), and 200 μL of normal culture medium was added to another 21 wells (control group). The medium was replaced with new medium once every 48 h. Each day, three wells of cells were taken and 20 μL of 3-(4,5-dimethylthiazol-2-yl)-2,5-diphenyltetrazolium bromide (MTT) was added to each well. After 4 h, the culture solution in the wells was aspirated. Then 150 μL of DMSO was added, followed by oscillation for 10 min. The optical density of the mixture was measured at a wavelength of 490 nm. A blank well was used as a control.

### Determination of ALP activity

BMSCs were inoculated into a 96-well plate at a density of 2,000 cells/well. In the experimental groups, after cell adherence, BMSCs were transfected by Ad-hBMP2-GFP (MOI = 100), followed by routine culture. In the control group, BMSCs were not transfected by Ad-hBMP2-GFP. On the 3rd, 6th, 9th, and 12th day, the supernatant of each well was removed and the wells were washed with PBS. Then 100 μL of 0.1% Triton X-100 was added to each well for overnight cleavage (4°C). The alkaline phosphatase (ALP) activity was determined using ALP kits (Stratagene Corp., California, USA) according to the manufacturer’s instructions. Using continuous monitoring methods (3 min), the absorbance change per minute (ΔA/min) was detected at a wavelength of 405 nm. The actual concentration of ALP (U/L) was calculated according to the following formula: U/L = ΔA/min × 2,757.

### Scanning electron microscope observation

After 3 days in culture, the cell-and-scaffold-composite specimen was taken out. After washing three times with PBS, 2.5% glutaraldehyde was used for fixation at room temperature, followed by graded dehydration with 60%–100% ethanol and replacement with isoamyl acetate. Then the specimen was dried at critical point temperature, followed by surface coating with gold and scanning electron microscope (SEM) observation.

### Ectopic osteogenesis experiments *in vivo*

Nude mice were bought from the laboratory of the animal center at the Chinese Medical Sciences University (8 weeks, average weight: 22.5 g). The left side of the femoral muscle was used as the experimental side (Ad-GFP-hBMP was added), and the right side was used as the control side (only normal growth and nutrient solutions were added). Animal responses were generally observed after transfection into the femoral muscles of nude mice. X-ray films were taken after 12 weeks to observe the ectopic osteogenesis effect. Then these mice were killed and samples were obtained for hematoxylin and eosin (HE) staining.

### X-ray examination

At the 4th, 8th, and 12th week after surgery, X-ray examination (normotopia projection, 40 kV, 50 mA, 0.2 s, 60 cm projection distance) was conducted on the bilateral ulna and radius of the rabbits.

### Observation on gross specimen and histological sections

At the 4th, 8th, and 12th week after surgery, six rabbits were executed. The ulna and radius in the area of bone defect were taken. The degradation of the implanted material, the combination with the host bone interface, and the reflection of the surrounding soft tissues were observed. The specimen was then treated by decalcification with 5% nitric acid for 72 h, followed by fixation with 4% formalin. After embedding in paraffin, slices with 10-μm thickness were continuously prepared and subjected to HE staining and observation.

### Statistical analysis

Data were expressed as $\overline{x}$ ± *s*. Statistical analysis was performed using SPSS 17.0 statistical software. Single factor ANOVA was conducted for comparison between different groups. *P* < 0.05 was considered as statistically significant.

## Results

### MTT detection

There was no significant difference in the results of MTT detection between the experimental group and control group (*P* > 0.05), suggesting that the medium immersing A-W-MGC/CS did not affect the growth and proliferation of BMSCs (Table 1).

**Table 1:** **MTT detection results at each time point in two groups (U/L,** ***n*** **= 7,** $\overline{x}$ **±** ***s*** **)**

**Group**	**1st day**	**2nd day**	**3rd day**	**4th day**	**5th day**	**6th day**	**7th day**
Experimental	0.23 ± 0.02	0.25 ± 0.03	0.38 ± 0.05	0.52 ± 0.08	0.87 ± 0.09	1.23 ± 0.19	1.24 ± 0.22
Control	0.24 ± 0.03	0.26 ± 0.02	0.41 ± 0.04	0.50 ± 0.04	0.92 ± 0.11	1.26 ± 0.25	1.25 ± 0.27

### Transfection

**Figure 1:**
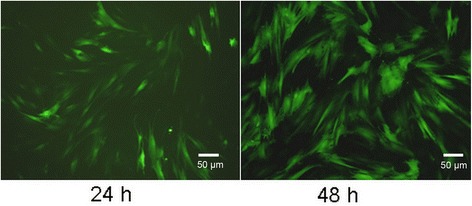
**Fluorescence microscopy of adenovirus transfected BMSCs (×100).**

**Figure 2:**
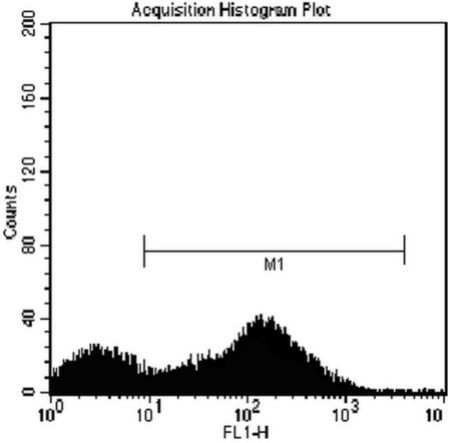
**Transfection rate of BMSCs (M1, 71.1** **%** **).**

At 24 h after adenovirus transfection, the BMSCs with fluorescence were visible. At 48 h, the number of BMSCs with fluorescence increased significantly (Figure 1). Results of flow cytometry showed that the transfection rate was 71.1% (Figure 2).

### ALP activity

Table 2 shows that the ALP activity of BMSCs in the experimental group and control group gradually increased with the prolonged experimental times, with significant differences among the different time points (*P* < 0.01). The ALP levels at each time point in the experimental group were higher than those in the control group. This result indicated that the transfected BMSCs could be transplanted into the body to promote osteogenesis in the bone defect area.

**Table 2:** **ALP activity at each time point in two groups (U/L,** ***n*** **= 7,** $\overline{x}$ **±** ***s*** **)**

**Group**	**3rd day**	**6th day**	**9th day**	**12th day**
Experimental	34.10 ± 2.96	72.34 ± 2.19	107.02 ± 2.18	136.03 ± 4.49
Control	4.93 ± 1.11	8.20 ± 0.18	15.87 ± 1.78	21.73 ± 2.75
*t*	24.43	77.22	85.58	57.47
*P*	0.00	0.00	0.00	0.00

### SEM observation results

**Figure 3:**
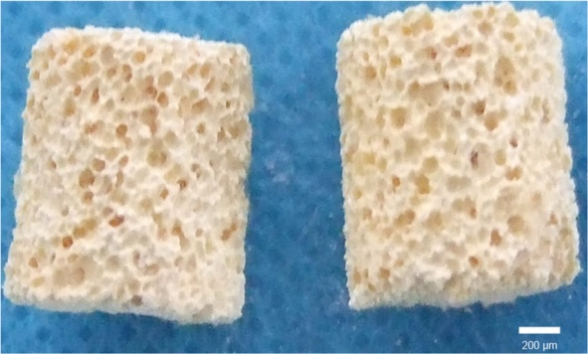
**Picture of A-W-MGC/CS scaffold.**

**Figure 4:**
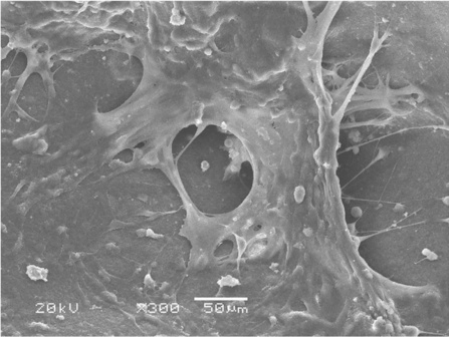
**Attachment and growth of BMSCs on scaffold (SEM).**

An image of the A-W-MGC/CS scaffold is shown in Figure 3. The results of the SEM observation of BMSCs on the scaffold are shown in Figure 4. Good attachment and growth of BMSCs were observed on the scaffold. Some cells showed fibroblast-like morphology, and some were circular or quasi-circular with flat cell bodies. Cells crossed the surface of the microbore, forming intercellular connections. Cells were connected into pieces, and a large amount of extracellular matrix was also observed.

### Heterotopic ossification experimental results

**Figure 5:**
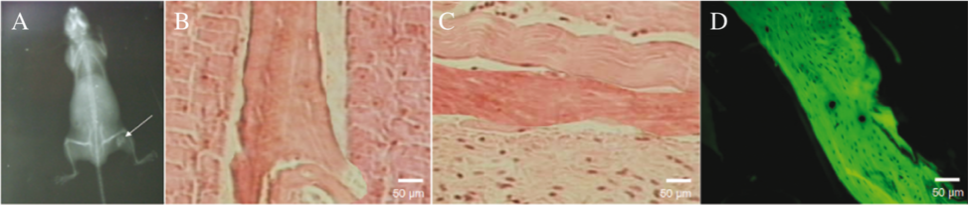
**X-ray slice and histological sections. (A)** X-ray slice of 12 weeks after BMSCs are transplanted into nude mouse indicates that high density calcified shadow is detected in experimental side, but not detected in control side (indicated by the *arrow*). **(B)** Histological section of experimental side with HE staining indicates that mature bone tissue can be detected between muscular tissues (×100). **(C)** Histological section of control side with HE staining indicates immature bone and cartilage tissue between muscular tissues (×100). **(D)** Bone tissue in control side appears as green fluorescence under the fluorescence microscope.

In the experimental group, X-ray films suggested that seven sides exhibited a high-density shadow, two sides exhibited a slightly high-density shadow, and one side did not show anything. The positive rate of ossification was 90%. In the control group, four sides exhibited a high-density shadow with uneven density, while six sides did not show anything, and the positive rate of ossification was 40% (Figure 5A). Histological observations indicated that more mature bone tissues were formed in the experimental group that appeared with green fluorescence under a fluorescence microscope, while some immature bone and cartilage were detected between muscle tissues in the control group (Figure 5B-D).

### X-ray examination results

**Figure 6:**
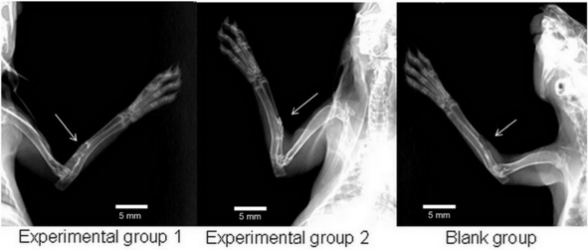
**X-ray examination results at the 4th week after surgery.** Bone calluses were observed in the bone defect area for experimental groups 1 and 2 while none was observed in the blank group (*arrows*).

**Figure 7:**
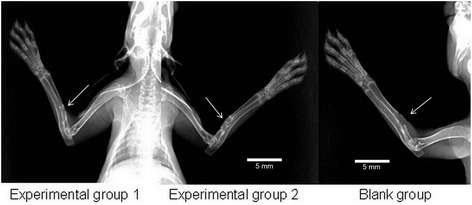
**X-ray examination results at the 8th week after surgery.** Continuous growth of new bone is observed in the bone defect area for experimental groups 1 and 2 while none was observed in the blank group (*arrows*).

**Figure 8:**
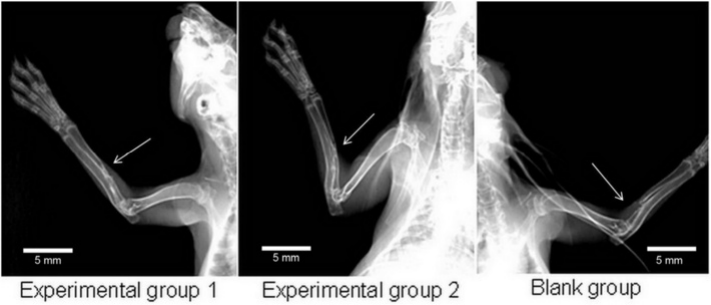
**X-ray examination results at the 12th week after surgery.** Bony callus in experimental group 1 has completely ossified while experiment group 2 showed an increase in density and number of the bony callus (*arrows*); in the blank group, the results were the same as those observed at the 8th week (*arrow*).

At the 4th week after surgery, in experimental group 1, continuous bone callus appeared at the center of the implanted material, and bilateral bony callus uniformly extended to the middle. The interface between the host bone and the tissue-engineered bone was fuzzy. In experimental group 2, there was continuous bone callus with a density lower than that of normal bone. In the blank group, no obvious bone callus was visible in the bone defect area (Figure 6). At the 8th week, in experimental group 1, there was continuous recovery of new bone cortex in the bone defect area, with blocked marrow cavity. The density of new bone was significantly lower than that of the host bone. In experimental group 2, continuous bone callus appeared in the region near the ulna in the bone defect area. In the blank group, there was no new bony callus in the bone defect area, and the fractured end of the bone was smooth (Figure 7). At the 12th week, in experimental group 1, the bony callus between tissue-engineered bone and the host bone has been completely ossified with unblocked marrow cavity. The density of the part near the ulna was especially high. In experimental group 2, the density and number of bony callus in the defect area were increased, with a smaller diameter than that of the host bone. In the blank group, the results were the same as those observed at the 8th week (Figure 8).

### Histological observation

**Figure 9:**
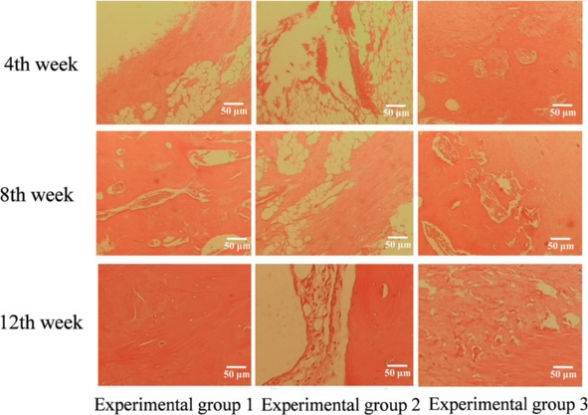
**Histological observation results at the 4th, 8th, and 12th week after surgery (HE staining, ×100).**

**Figure 10:**
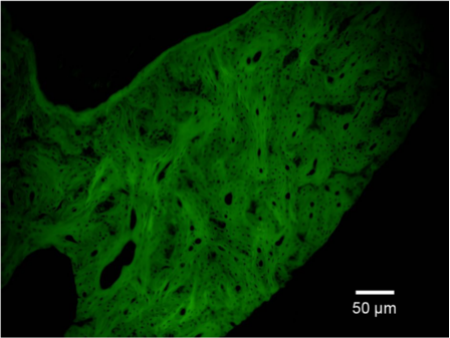
**Fluorescence microscopy of bone defect area (×100).**

The results of histological observation at the 4th, 8th, and 12th week after surgery are shown in Figure 9. At the 4th week after surgery, in experimental group 1, the tissue-engineered bone had been degraded into a mesh-shaped structure with the generation of a large number of chondrocytes in the bone defect area containing a small amount of bone matrix. In experimental group 2, many chondrocytes were observed in the area near the ulna and the fractured end. In the blank group, there were fibrous tissues and a large number of lymphocytes. At the 8th week, in experimental group 1, there were a large number of mature woven bones with a small amount of remaining mesh-shaped structures. A small amount of cartilage matrix and a considerable amount of bone trabecula were visible. In experimental group 2, there were mature woven bones in the area near the ulna and the fractured end. In the blank group, mainly fibrous tissues were seen. At the 12th week, in experimental group 1, there were mature bone tissues with more bone trabecula. In experimental group 2, part of the cartilage matrix was still visible. The amount of bone trabecula was less than that observed in experimental group 1. In the blank group, there were a few chondrocytes and cartilage tissues in the bone defect area near the host bone. The remaining parts were still filled with fibrous tissues. In addition, under a fluorescence microscope, the new bone in the bone defect area in experimental group 1 emitted green fluorescence (Figure 10), and no green fluorescence was observed in experimental group 2 or the blank group.

## Discussion

As seed cells, BMSCs have stable characteristics and can be easily obtained and amplified. In addition, they have the potential of multi-directional differentiation and have been widely used in tissue engineering. The cytokine BMP2 can promote bone defect repair and fracture healing, thus exhibiting an osteoinductive activity [10-12]. The tracing factor GFP is a reporter gene in the study of gene transfection, which has stable photoluminescent properties and is convenient for detection. It can be used for live-cell labeling and *in vivo* tracing [13-15]. As a transfection media, adenovirus vectors have high transfection efficiency. Exogenous genes can be expressed at high levels without being integrated into the host genome, thus ensuring the safety of the transfection [16]. In this study, Ad-hBMP2-GFP was transfected into rabbit BMSCs in *vitro*. According to previous experimental results [17], the MOI was set as 100. The BMP2 gene could be efficiently transfected into the cells (full view of green fluorescence could be observed 48 h after transfection, and the transfection rate was close to 71%). The products of BMP2 expression were secreted into the peripheral environment to feedback and promote cell proliferation and osteoblastic phenotype expression. The results of ALP activity detection showed that BMSCs were well differentiated. Histological observations found that the new bone was uniformly generated at the transverse section of the bone defect area. This result indicated that there was ossification not only in permeated autologous BMSCs on the outer surface of the implanted material but also in cells carried by the implanted material. This ossification can greatly accelerate the bone defect repair.

Ideal extracellular matrix materials in bone tissue engineering should satisfy the following requirements [18-20]. (1) Good biocompatibility and surface activity. In this study, A-W-MGC/CS was used as a carrier to construct tissue-engineered bone. A-W-MGC/CS was prepared by Professor Zhou Dali from the Department of Inorganic Non-metallic Materials of Sichuan University (Chengdu, China) using the sol–gel method, but not the traditional melting method. This material had a nano-crystalline structure, which could guarantee the adjustability of the microenvironment on the surface. As an excellent bone tissue engineering scaffold developed in these past few years, A-W-MGC/CS possessed good biocompatibility, bone conductibility, bone inducibility, and mechanical strength. More importantly, it functioned in directly inducing the differentiation of BMSCs into osteoblasts. The *in vivo* dissolution of AW-MGC/CS could increase the concentration of Ca^2+^, thereby enhancing the gene expression of transforming growth factor-β1, improving the activities of osteoblasts, and promoting their differentiation. Meanwhile, the osteoblasts could directly calcify on the bone surface. The osteoid among the surface and among cells would directly calcify, and did not form layers of connective tissue membranes. The degradation rate of AW-MGC/CS was lower than that of β-TCP, thus avoiding the phenomenon of excessive degradation-induced “powder residue stasis,” and the Ca-P-rich layer generated during this degradation could be directly used by the osteoblasts to form bones.

*In vitro* SEM observations showed that the cells had good adherence and growth with uniform distribution, regular form, and good attachment to the surface of the implanted material. These observations confirmed the feasibility of A-W-MGC/CS as a carrier of seed cells for bone tissue engineering. *In vivo* experiments also proved that A-W-MGC/CS possessed a high ability of ossification and bone defect repair. (2) Excellent biodegradability. Histological observations showed that at the 4th week after implantation, in experimental group 1, the implanted material has degraded into a mesh-shaped structure, and the newly formed osteoid tissues perforated in it. Cartilage-like cell clusters were distributed in the implanted material. In experimental group 2, many chondrocytes were found in the area near the ulna and the fractured end, which may be related to the participation of osteoblasts at the ulnar periosteum and the fractured end in bone repair. At the 12th week, the implanted material has virtually degraded completely. (3) Porousness and high porosity. The carrier had a three-dimensional porous structure, and the pores were connected to each other with uniform distribution. The aperture was 100–500 μm with a porosity of 80%–90%. In this study, SEM examinations showed good adherence and growth of cells on the implanted material. Histological observations showed that in experimental group 1, the new bone was uniformly distributed on the transverse section of the bone defect area, suggesting that the growth of new bone was uniform. After implantation of the tissue engineered bone, the seed cells on the implanted material not only proliferated well and secreted more extracellular matrix, but also retained the basic biological characteristics. These cells absorbed nutrients from the blood and extracellular fluid in the area of surgery. After a period of time, the surrounding capillaries grew into the pores in the implanted material and exchanged nutrients and metabolites with the seed cells. As a result, the cells could continue to proliferate and secrete large amounts of extracellular matrix. Histological observations showed that osteoid tissues were present in the implanted material with newly formed vessels. Observations of tissue sections found that the growth of new bone on the transverse section of the bone defect area was uniform with no decrease in osteogenic ability due to cell death in the implanted material. (4) Plasticity and a certain degree of mechanical strength. The scaffolding material could be molded into a variety of complex configurations according to the foam body shape. The mechanical strength of the scaffold has been validated in early experiments, with no appearance of second fracture in *in vivo* experiments. (5) Osteoconductivity and osteoinductivity. In this study, continuous bone callus appeared early in the bone defect area. Histological observations showed that osteoid tissues were also present in the central bone defect area. MTT results found that A-W-MGC/CS had good biocompatibility, no cytotoxicity, and did not affect the growth and proliferation of cells. In addition, during the whole stage after implantation of the scaffolding material, no signs of acute inflammation were detected in the bone defect area except for the infiltration of a small number of lymphocytes at the 4th week. This observation indicated that the scaffolding material exhibited high biocompatibility without causing immune rejection to the body.

## Conclusions

The AW-MGC/CS-constructed tissue-engineered bone, combined with Ad-GFP-hBMP-transfected BMSCs, exhibited better osteogenic repairing abilities towards bone defects. These results indicated that the scaffold applied to treat bone defects could affect osteoconductivity.
